# Comparison of short-term efficacy analysis of medium-rectal cancer surgery with robotic natural orifice specimen extraction and robotic transabdominal specimen extraction

**DOI:** 10.1186/s12893-023-02216-y

**Published:** 2023-11-08

**Authors:** Shan-ping Ye, Wei-jie Lu, Dong-ning Liu, Hong-xin Yu, Can Wu, Hao-cheng Xu, Tai-yuan Li

**Affiliations:** Department of General Surgery, The First Affiliated Hospital of Nanchang University, No. 17 Yongwaizheng Street, Nanchang, 330006 Jiangxi Province China

**Keywords:** Medium-rectal cancer, Robot-assisted, Natural orifice specimen extraction surgery

## Abstract

**Background:**

With the development of minimally invasive technology, the trauma caused by surgery get smaller, At the same time, the specimen extraction surgery through the natural orifice is more favored by experts domestically and abroad, robotic surgery has further promoted the development of specimen extraction surgery through the natural orifice. The aim of current study is to compare the short-term outcomes of robotic-assisted natural orifice specimen extraction (NOSES ) and transabdominal specimen extraction(TRSE ) in median rectal cancer surgery.

**Methods:**

From January 2020 to January 2023, 87 patients who underwent the NOSES or TRSE at the First Affiliated Hospital of Nanchang University were included in the study, 4 patients were excluded due to liver metastasis. Of these, 50 patients were in the TRSE and 33 patients in the NOSES. Short-term efficacy was compared in the two groups.

**Results:**

The NOSES group had less operation time (P < 0.001), faster recovery of gastrointestinal function (P < 0.001), shorter abdominal incisions (P < 0.001), lower pain scores(P < 0.001). lower Inflammatory indicators of the white blood cell count and C-reactive protein content at 1, 3, and 5 days after surgery (P < 0.001, P = 0.037). There were 9 complications in the NOSES group and 11 complications in the TRSE group(P = 0.583). However, there were no wound complications in the NOSES group. The number of postoperative hospital stays seems to be same in the two groups. And there was no significant difference in postoperative anus function (P = 0.591).

**Conclusions:**

This study shows that NOSES and TRSE can achieve similar radical treatment effects, NOSES is a feasible and safe way to take specimens for rectal cancer surgery in accordance with the indication for NOSES.

## Background

A major concern to the safety of the public’s health is the prevalence of colorectal cancer, which is the third most prevalent cancer and has a very high fatality rate [1, 2]. There are multiple treatments for rectal cancer, and surgery remains one of the most important ways. Laparoscopic surgery as a minimally invasive technique for the treatment of colorectal has been confirmed by many studies to ensure its safety and reliability [3, 4]. Laparoscopic surgery has also been widely used in clinical [5, 6]. So far, NOSES, as an emerging minimally invasive technology, has caused heated discussions in the minimally invasive surgical community [7–9], especially in rectal surgery [10–12], the emergence of NOSES, which solves the problems caused by incisions in traditional surgery, improves the mental health of patients, and has good short-term efficacy [13]. In recent years, the popularity of robotic colorectal cancer surgery has been rising, and the concepts of radical treatment, precision, and minimally invasive have been continuously refined, and NOSES surgery, as an emerging minimally invasive technology, has further reduced the impact of surgical trauma on the body, eliminated abdominal scar incision, and avoided complications related to abdominal wall incision, and has been widely used and carried out [14]. In addition to the benefits of patients, the high-definition lens of the robotic surgery system and the flexible robotic arm greatly remove the trembling of the operator’s hand, improve the flexibility and accuracy of the operator’s operation, and are more conducive to challenging operations in narrow spaces. Compared with laparoscopy, the robotic surgical system has great advantages in some aspects, such as postoperative patient urination function, sexual function, surgical complications [12, 15, 16]. Robotic surgical systems, combined with NOSES, may bring greater benefits to patients. Therefore, we reviewed the clinical data of 87 patients who underwent robot-assisted radical rectal resection from January 2020 to January 2023, who underwent conventional abdominal specimen extraction and radical rectal cancer resection through natural orifice. Then the short-term efficacy of different specimen retrieval routes is compared to explore the safety and feasibility of NOSES in robotic radical rectal cancer surgery.

## Methods

In the current study, we collected the clinicopathological data of median rectal cancer surgery patients who underwent robotic surgery at the First Affiliated Hospital of Nanchang University between January 2020 and January 2023. Then analyzed. A total of 87 patients with median rectal cancer underwent robotic surgery, 4 patients were excluded due to liver metastasis, and a total of 83 patients met the criteria, including 33 cases in the noses group and 50 cases in the abdominal specimen group. The study protocol was approved by the institutional review board of The First Affiliated Hospital of Nanchang University. The study compliance with the Helsinki Declaration. Written informed consents were obtained from all of the patients.

Inclusion Criteria: (1) age greater than 18 years and below 80 years old; (2) Endoscopic biopsy confirmed primary colon adenocarcinoma; (3) According to the preoperative examination and intraoperative observation, it was confirmed that there was no distant metastasis; (4) American Society of Anesthesiologists (ASA) score I, II, or III; (5) Sign informed consent. (6) According to imaging examination, colonoscopy, intraoperative and postoperative pathology, it was confirmed that the tumor was located in the middle rectum;

Exclusion criteria: (1) concurrent with other malignant tumors; (2) Cases of emergency surgery due to bleeding, obstruction, and perforation; (3) Transit laparotomy; (4) Incomplete data or missing follow-up data; (5) Patients with preventive stoma or patients with ostomy for other reasons.

### Surgical procedures

**Fig. 1:**
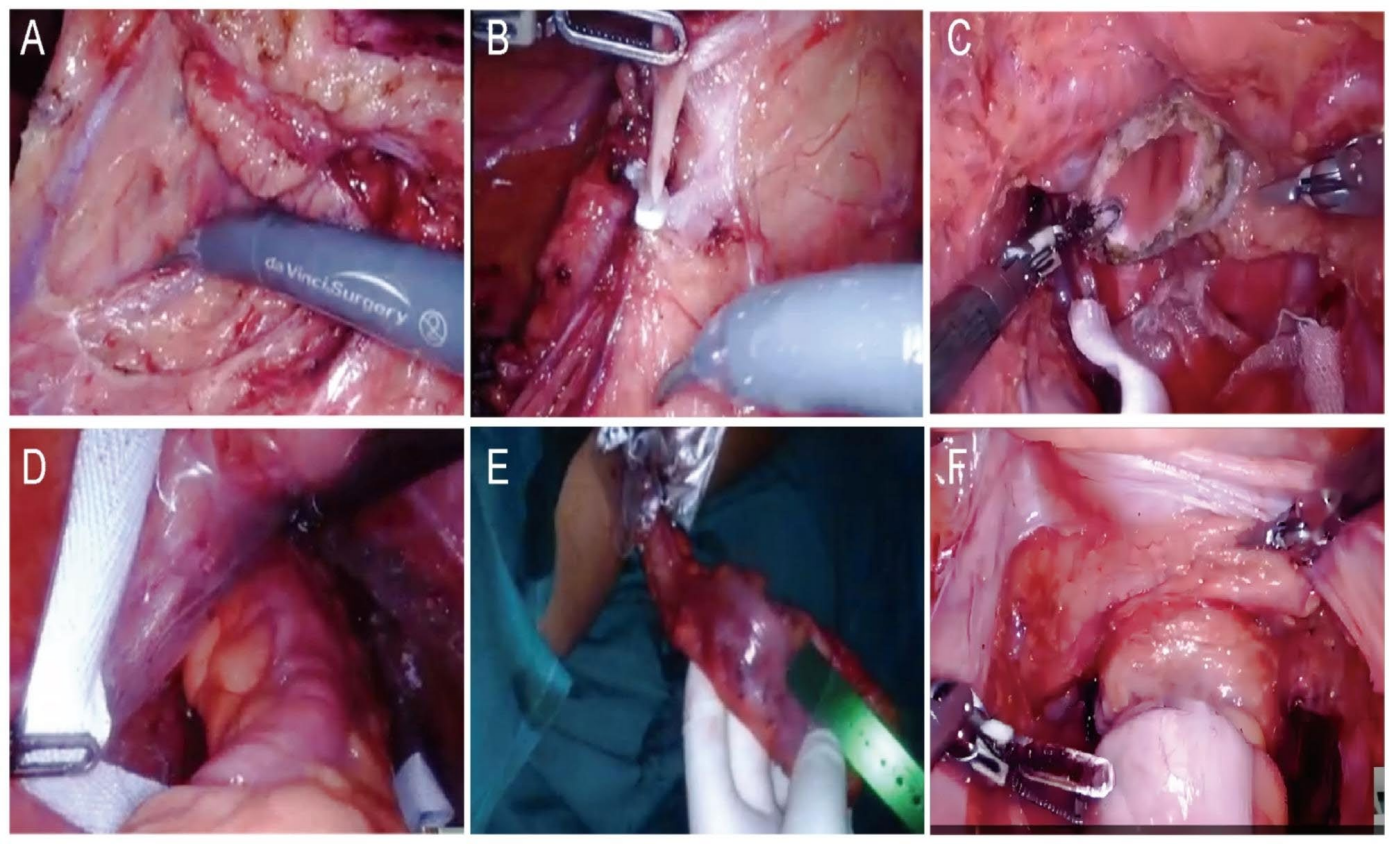
Key surgical steps of NOSES (A–F). (**A**) Expose the inferior mesenteric artery. (**B**) Ligate the inferior mesenteric artery. (**C**) A sterile protective sleeve was placed into the anus to establish sterile access. (**D, E**) The proximal rectum is dragged out through the protective sleeve. (**F**) Suturing the anastomosis

The patients’ position and trocar position can be referred to our previous study [17]. After successful anesthesia, abdominal exploration is performed to determine whether there are metastases and other conditions, and digital rectal examination is combined with palpation of abdomen to determine the location and size of the tumor. Expose the inferior mesenteric artery, clean the lymph nodes at the root of the vessel, ligate and separate the inferior mesenteric artery, free sigmoid colon and rectum, free the entire mesentery, bare the rectum, cut the closure 2 cm from the lower edge of the tumor to break the rectum. We will pull the severed intestinal tube towards the anus to determine if its length is sufficient. If the intestinal tube is not long enough, we will free the spleen and colon. Fully and softly extend the anus, and rinse the rectal lumen with iodophors through the anus. A protective sleeve is inserted through the helper hole, and the protective sleeve is dragged out of the anus through the rectum, and the proximal rectum is dragged out through the protective sleeve, and the terminal ileum is cut 10 cm above the tumor. The anvil is placed into the sigmoid colon stump, clamped with toothed forceps, and sent to the abdominal cavity through the protective sleeve. Finally, the stapler is inserted through the anus, then complete the end anastomosis, and the iodophors injection test checks whether there is leakage in the anastomosis and sutures the anastomosis. The part of the surgical process of NOSES were shown in the Fig. 1.

### Parameters of observation and evaluation parameters

The general demographic data of the patient are as follows: age, sex, body mass index (BMI). The surgical parameters of the patients were as follows: American Society of Anesthesiologists (ASA) score, total time to surgery, intraoperative blood loss, white blood cell count, and C-reactive protein (CRP) were used to assess the postoperative inflammatory response, postoperative activity time, postoperative ventilation time, and postoperative complications were recorded by Clavien-Dindo classification, Visual Analogue Scale (VAS) scores, the Wexner Incontinence Score assesses function 3 months after surgery, postoperative hospital stay.

### Statistical analysis

All statistical analyses used SPSS26.0 and were tested for normality for all parameters, with measurements that conforming to the normal distribution expressed as mean ± SD and non-normally distributed data expressed as median and range, using either the independent sample t-test or the Mann-Whitney U test, respectively. Counting data is expressed as frequency and percentage, using the χ2 test or the Fisher exact probability method. P < 0.05 was considered statistically significant.

## Results

### Clinical baseline characteristics

Table 1 shows that the sex, age, BMI, preoperative white blood cell count, preoperative C-reactive protein level, tumor distance from the anus, tumor size, TNM stage, and ASA score were compared in the study, then there was no significant difference in clinical baseline characteristics between the two groups (p > 0.05).

**Table 1:** Comparison of baseline data between NOSES and TRSE group

	NOSES (n = 33)	TRSE (n = 50)	p
Gender			0.389
male	18(54.5%)	32(66.7%)	
female	15(45.5%)	18(33.3%)	
age	58.3 ± 10.98	57.88 ± 10.67	0.870
BMI	22.17 ± 2.53	23 ± 2.12	0.111
CRP	4.87 ± 6.18	7.95 ± 13.64	0.229
WBC	5.74 ± 1.47	5.72 ± 1.47	0.964
Distance of tumor and anal, cm	7.9 ± 1.85	7.78 ± 1.85	0.774
Diameter of neoplasm, cm	3.7 ± 0.82	4 ± 1.11	0.102
pTNM			0.336
I	9(27.3%)	8(16%)	
II	11(33.3%)	15(30%)	
III	13(39.4%)	27(54%)	
ASA			0.725
II	10(30.3%)	17(34%)	
III	23(69.7%)	33(66%)	

### Comparison of perioperative indexes between TRSE group and NOSES group

**Fig. 2:**
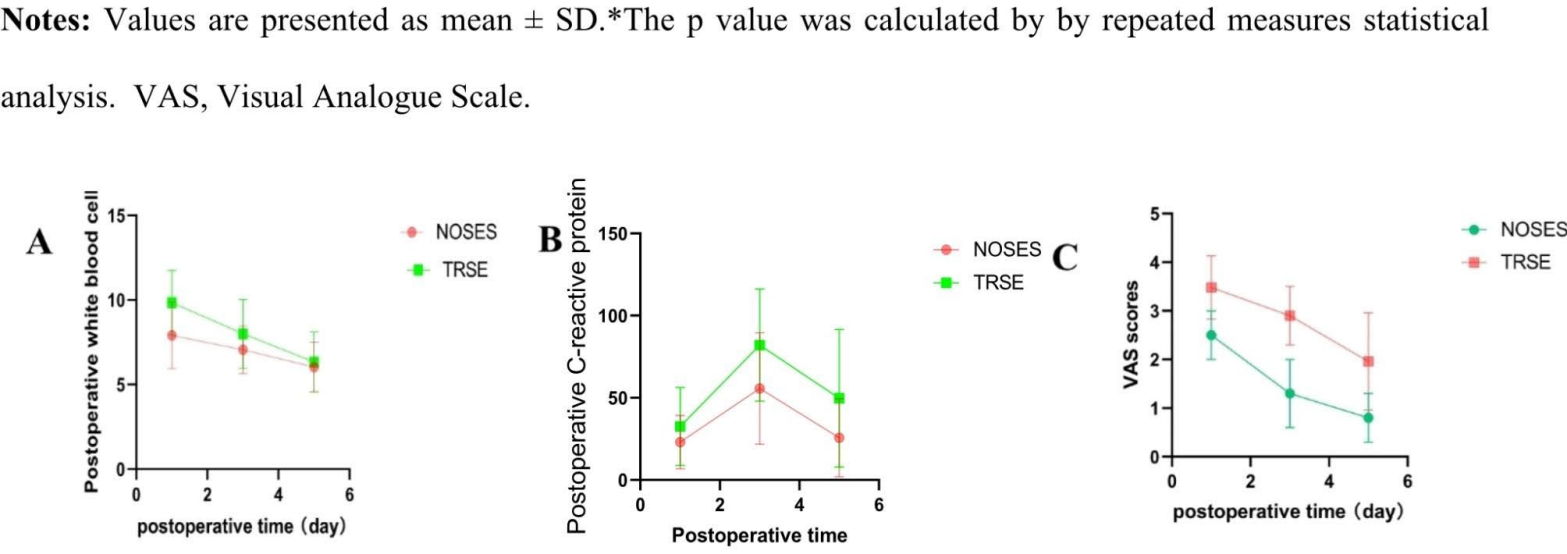
Comparison of Perioperative Indexes Between two groups of patients. (**A**). White blood cell scores between two groups; (**B**) C-reactive protein scores between two groups (**C**) VAS scores between two groups;

These table presents comparison of the perioperative data of the two groups, the operation time of the two groups was slightly different (143.33 ± 30.71 min in the NOSES group vs. 166.50 ± 30.28 min in the TRSE group P = 0.001), and the intraoperative blood loss was similar (133.80 ± 62.33 ml in the TRSE group VS 106.67 ± 61.93ml in the NOSES group, P = 0.06). But the gastrointestinal recovery function in the NOSES group was better than that in the TRSE group (66.70 ± 6.69 h in the TRSE group VS 58.48 ± 4.56 h in the NOSES group, P < 0.01), and the length of abdominal incision was significantly shorter than that in the TRSE group (11.9 ± 0.6 cm in the TRSE group vs. 4.9 ± 0.2 cm in the NOSES group, P < 0.001). In terms of postoperative recovery indicators, the number of days of postoperative hospital stay was the same in the TRSE group and the NOSES group (P = 0.470 (Table 2). However, pain scores in the NOSES group were better than in the RARS group (P < 0.001), and significantly fewer patients required additional analgesics than in the TRSE group. In terms of surgical stress, we compared the white blood cell count and C-reactive protein content of the two groups at 1, 3 and 5 days after surgery, and the inflammatory indicators of the NOSES group were lower than those in the TRSE group (P = 0.005, P = 0.002) (Table 3; Fig. 2 ). In terms of postoperative complications, there were 9 complications in the NOSES group and 11complications in the TRSE group. It is worth mentioning that there were no complications in the wounds of the NOSES group. And there was no significant difference in postoperative anus function (P < 0.001, Table 4). No bacteria were cultured in the peritoneal lavage fluid of both NOSES and TRSE groups of patients.

**Table 2:** Comparison of Postoperative Conditions between NOSES group and TRSE group

Outcome	NOSES(n = 33)	TRSE (n = 50)	p
Operative time, mean (SD), min	143.33 ± 30.71	166.50 ± 30.28	< 0.001
Estimated blood loss, mean (SD), ml	106.67 ± 61.93	133.80 ± 62.33	0.060
1st flatus, mean (SD), hour	58.48 ± 4.56	66.70 ± 6.69	< 0.001
1st motion, mean (SD), hour	23.6 ± 1.8	30.58 ± 3.16	< 0.001
1st oral feeding, mean (SD), hour	74.48 ± 3.9	74.2 ± 3.9	0.746
abdominal incision, mean (SD), cm	4.9 ± 0.2	11.9 ± 0.6	< 0.001
Postoperative hospital stay, mean (SD), d	11.78 ± 7.31	10.74 ± 5.80	0.470
Harvested lymph nodes, n (%)			0.283
≤ 12	10(30.3)	10(20)	
>12	23(69.7)	40(80)	
Postoperative complication, n (%)	9(27.3)	11(22)	0.583
Anastomotic leakage	4(12.13)	3(6)	
Ileus	0	1(2)	
Wound-related	0	2(4)	
Urinary retention or infection	1(3.03)	1(2)	
Pulmonary infection	2(6.06)	2(4)	
Others	2(6.06)	2(4)	

**Notes**: Values are presented as mean ± SD or n (%)

**Table 3:** Comparison of postoperative stress response and pain condition of patients between NOSES group and TRSE group

	NOSES(n = 33)	TRSE (n = 50)	p
Postoperative white blood cell, mean (SD), count /l			0.001
Day 1	7.9 ± 1.97	9.85 ± 1.9	
Day 3	7.06 ± 1.41	8.01 ± 2.04	
Day 5	6.03 ± 1.48	6.33 ± 1.77	
Postoperative C-reactive protein, mean (SD), mg/l			0.037
Day 1	23.1 ± 16.27	32.5 ± 23.84	
Day3	55.7 ± 33.9	82 ± 34.2	
Day 5	25.68 ± 23.75	49.69 ± 41.86	
VAS scores, mean (SD)			< 0.001
Day 1	2.5 ± 0.5	3.48 ± 0.65	
Day 3	1.3 ± 0.7	2.9 ± 0.6	
Day 5	0.8 ± 0.5	1.96 ± 1.0	

**Table 4:** Comparison of Wexner scores between noses group and TRSE group

Type of incontinence	noses(n = 33)	TRSE (n = 50)	p
Solid	1(0–3)	1(0–3)	0.591
Liquid	1(0–3)	1(0–3)	
Gas	1(1–2)	1(1–2)	
Wears pad	1(0–2)	1(0–1)	
Lifestyle alteration	1(0–2)	1(0–1)	
Total score	6(4–9)	6(4–10)	

## Discussion

With the continuous development of minimally invasive technology, radical rectal cancer surgery has coexisted from traditional laparotomy to laparoscopic surgery [18, 19], and now robotic surgery [20]. In recent years, the number of reports of colorectal cancer resection of robotic natural orifice specimen extraction has increased [21]. At the same time, there is a variation of NOSES [9]. The surgical treatment of colorectal cancer is gradually developing in the direction of minimally invasive surgery, which is the current trend in the field of surgery. Conventional laparoscopic rectal cancer surgery involves removing the tumor specimen through an incision of 5 to 7 cm in the abdomen [22, 23]. With the development of surgical technology and the deepening of minimally invasive concepts, a new surgical technology (NOSES) to avoid abdominal auxiliary incision. To take specimens through the natural orifice has gradually become a research hotspot [24]. The safety and postoperative effect of surgery are topics of great concern [25–27],what’s more, the safety of surgery is a prerequisite for a new technology operation. In this study, the NOSES group was performed by the same surgeon to avoid the drift of the study data caused by the learning curve [28], and the surgeon had rich surgical experience. The procedure strictly adheres to the principle of aseptic surgery, uses a sterile protective sleeve to remove the specimen, then sterilizes it in time, finally rinses the abdominal cavity with plenty of normal saline. In addition, the results of this study showed that the bacterial culture of postoperative abdominal pelvic lavage fluid in the NOSES group was negative. In terms of postoperative complications, there was no significant difference in postoperative intraoperative infection between the NOSES group and the abdomen group. In addition, the average operation time of the two groups was slightly different, and the author considered that the time for gastrointestinal reconstruction was shortened and the amount of bleeding was similar. In terms of the tumor-free principle, we found no significant difference in the number of lymph node dissections between the two groups. This is due to the robotic surgical system’s wider field of view and flexible robotic arms. These results further confirm that robotic colorectal cancer resection through natural orifice specimens has a surgical efficacy that is not inferior to robotic-assisted rectal cancer resection [29, 30]. The short-term efficacy of surgery is an important indicator to evaluate the quality of robotic NOSES surgery for colorectal cancer. Because the length of the abdominal wall incision in the robotic NOSES surgery is significantly shorter than the abdomen group, the damage to the abdominal wall is reduced, the postoperative pain of the patient is also significantly reduced, the additional analgesic required is also less, at the same time the patient can get out of bed early. Therefore, the recovery time of gastrointestinal function in the NOSES group is earlier than the abdomen group. NOSES surgery has no incision in the abdomen, doesn’t destroy the integrity of the abdominal wall and avoid the occurrence of near and long incision dehiscence and incision hernia; Similarly, we compared leukocyte markers and C-reactive protein levels at 1, 3, and 5 days after surgery between the two groups. A research indicated [31]that the stress response to surgery may promote the growth of pre-existing micrometastasis or may trigger tumor spread. The inflammatory indexes of the NOSES group were lower than those in the abdomen group, which indicated that the NOSES group had less interference with the patient’s body and a more obvious advantage of minimally invasive surgery. Of course, there are some limitations to this study. First, it was retrospective research, it has the unavoidable selective bias. Secondly, because it is a single-center study, it is limited by its small size and has insufficient sample size. In order to reduce differences in background or surgical skills between different surgeons, it can ensure that all surgeries in this study were performed by a team of professionals led by the same surgeon. To this end, the center is carrying out a multicenter prospective randomized controlled study of robotic NOSES, which is believed to provide a higher level of evidence for robotic NOSES, so as to better guide the surgical treatment of colorectal cancer. In addition, the postoperative follow-up time in this study was short, and the long-term survival outcomes and disease-free survival of the two groups were not studied.

## Conclusions

Robotic NOSES surgery for colorectal cancer is a safe and feasible minimally invasive technique, and has a shorter abdominal incision, less pain, less surgical stress, faster postoperative motion, more conducive to the recovery of intestinal function. For suitable colorectal cancer patients, this technology can be further promoted. Of course, NOSES surgery is still developing, and the author also hopes that surgical experts across the country and even the world can strictly follow the guidelines to perform noses surgery for suitable patients and obtain greater benefits for patients.

## Data Availability

Access to the database can be obtained from the corresponding author on reasonable request.

## Abbreviations

ASA: American Society of Anesthesiologists
BMI: body mass index
CRP: C-reactive protein
NOSES: natural orifice specimen extraction surgery
SD: Standard Deviation
TNM: tumor-node-metastasis
TRSE: transabdominal specimen extraction
